# Environmental enrichment interaction for laboratory beagle dogs used in research

**DOI:** 10.29374/2527-2179.bjvm006323

**Published:** 2024-02-16

**Authors:** Anna Julia Bessa Fernandes, Fernanda da Silva Freitas Campos, Gabriella Santos Oliveira, Priscila Cardim Oliveira, Debora Azevedo Borges, Ivan de Alamar Pedrosa, Fabio Barbour Scott

**Affiliations:** 1 Veterinarian, Programa de Pós-Graduação em Ciências Veterinárias (PPGCV), Departamento de Parasitologia Animal (DPA), Instituto de Veterinária (IV), Universidade Federal Rural do Rio de Janeiro (UFRRJ). Seropédica, RJ. Brazil.; 2 Undergraduate in Veterinary Medicine, IV, UFRRJ. Seropédica, RJ, Brazil.; 3 Veterinarian, Autonomus, Rio de Janeiro, RJ, Brazil.; 4 Veterinarian, DSc. PPGCV, Departamento de Parasitologia Animal DPA. IV, UFRRJ. Seropédica, RJ. Brazil.; 5 Zootecnist, Autonomus, Rio de Janeiro, RJ, Brazil; 6 Veterinarian, DSc. DPA, IV, UFRRJ. Seropédica, RJ, Brazil.

**Keywords:** canine welfare, canine behavior, environmental stimulus, experimental laboratory, bem-estar animal, comportamento canino, estímulo ambiental, laboratório experimental

## Abstract

Experimental animal facilities can have a negative impact on the well-being of animals owing to confinement. To mitigate this, environmental enrichment (EE) is implemented confinement. The purpose of EE is to enhance the complexity of an animal’s natural environment. The objective of this study was to identify the types of EE most enjoyed by dogs used in experimental research and housed in individual kennels. A total of six adult Beagle dogs, housed at the Laboratório de Quimioterapia Experimental em Parasitologia Veterinária (LQEPV) at the Universidade Federal Rural do Rio de Janeiro (UFRRJ) were included in the study. The EE tools used included Petball® toys, a grassy outdoor area, interaction with other dogs and with a team member, a “pool” made of plastic bottles, and dog’s wet food ice cream. A team member assessed the usage of these tools every 5 min for a 30-min period, six times per day, one day per week, over the course of eight weeks. The study revealed that the grass area was the tool most commonly used for physical enrichment, accounting for 58% of the occurrences (*p* < 0.05). Social and food enrichment were enjoyed in second and third place, with 23% and 19% of occurrences, respectively. that the study findings suggest that dogs housed in individual kennels enjoy engaging in their natural behaviors.

## Resumo

Os animais de biotério podem viver em situações que afetem o seu bem-estar, como o confinamento. Um dos métodos que buscam para reduzir os impactos negativos é o Enriquecimento Ambiental (EA), o qual objetiva a promoção da complexidade do ambiente dos animais. Foi possível, com esta pesquisa, atingir o objetivo de identificar quais tipos de EA são mais aproveitados por cães utilizados em pesquisas experimentais que viviam em canis individuais. Foram utilizados seis cães adultos, Beagles, oriundos do Laboratório de Quimioterapia Experimental em Parasitologia Veterinária (LQEPV) da Universidade Federal Rural do Rio de Janeiro (UFRRJ). Como ferramentas de EA foram utilizados brinquedos Petball®, área de grama ao ar livre, interação com outros cães e com uma pessoa da equipe, “piscina” de garrafas plásticas e sorvete de ração úmida. Um membro da equipe avaliou o uso a cada 5 minutos, por período de 30 minutos (6 avaliações) por dia, um dia por semana ao longo de 8 semanas. O estudo revelou que a ferramenta mais usufruída pelos cães foi a área de grama através do enriquecimento físico, quantificado em 58% de ocorrência (p<0,05). Os enriquecimentos sociais e alimentar, foram usufruídos em segundo e terceiro lugar, com 23% e 19% de ocorrência, respectivamente. Pode-se concluir que os cães mantidos em canis individuais individualizados mais necessitam exercer seu comportamento natural específico, para que seu grau de bem-estar seja mantido em nível satisfatório e em consequência sua saúde mental.

## Introduction

The science of animal welfare (AW) has gained attention and interest from a growing segment of society, demonstrating an increased concern for improving the quality of life for animals. When animals are subjected toa behavioral limitations, such as those used in experimental laboratories, it becomes necessary to provide an environment that mimics natural conditions to enhance their welfare (Brasil, 2018).

AW is directly associated with the concept of the five freedoms, which states that every animal should: be free from hunger and thirst; be free from discomfort and live in an environment appropriate for the species; be free from fear and distress; and be free to express its natural behaviors (Great Britain, 2003). It is important to note that housing conditions that fail to meet the social or physical needs of laboratory animals can potentially lead to alterations in their physical and mental health, which may affect research outcomes, owing to altered physiological parameters caused by cortisol and stress (Assis, 2018).

One method employed to enhance animal welfare in experimental facilities is the use of environmental enrichment (EE) techniques, which can be categorized as social, cognitive, physical, sensory, or nutritional. This practice aims to promote behavioral diversity, increase the frequency of normal standard behaviors, encourage positive utilization of the environment, foster natural adaptation to challenges, and reduce the occurrence of abnormal behavior (Young, 2013).

This study aimed to identify the type of EE preferentially used by beagle dogs housed in individual kennels at the LQEPV.

## Material and methods

### Experimental setup

The experiment was authorized by the Ethics Committee on the Use of Animals (CEUA) at the UFRRJ Seropedica Campus under protocol number 7007091219.

The study participants were six clinically healthy adult beagle dogs, three male and three female, ranging in age from two to eight years. The animals were housed at the Laboratório de Quimioterapia Experimental em Parasitologia Veterinária (LQEPV) da Universidade Federal Rural do Rio de Janeiro (UFRRJ). The animals were divided into two groups of three animals each: one group consisted of females (G1), and the other group consisted of males (G2). Each dog was kept in an individual kennel with an area of approximately 3 m^2^, including 1.5 m^2^ of covered area. The water and the commercial dry dog food bowls, as well as the animals’ beds were kept in the covered area. The dogs were free to use the open area as they pleased. Food was offered twice daily, and water was provided *ad libitum*.

The following EE tools were used: Pet Ball® toys filled with a mix of dry food (Must® Adult dog food, small and medium breeds, chicken flavor) and wet food (Kifera® for adult dogs, chicken flavor) placed indoors, a grassy outdoor area, interaction with dogs of the same sex and with a team member, polyethylene terephthalate (PET) bottle “pool” with a mix of dry and wet food, and ice cream made from a 1:1 ratio of wet food and water (Table 1). Prior to releasing the animals, a recreation kennel with an approximate area of 10 m^2^ was prepared with the aforementioned EE tools. The kennel consisted of a masonry structure with a roof serving as a shelter, a bed, a collective feeder, an automatic drinking fountain, and an uncovered grassy area.

**Table 1:** Environmental Enrichment Tools used and their descriptions.

**Tool**	**Description**	**EE type**	**Interaction**	**Image**
**PET Pool**	Rectangular wooden structure filled with several PET bottles without labels, lids, and seals, containing dry and wet food mixed beforehand and distributed among the bottles.	Physical; Food and Sensorial	Animal enters the pool; it rummages and tramples the bottles in search of food.	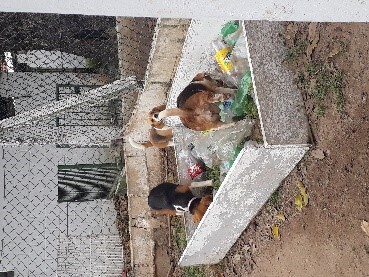
**Pet Ball®**	Spherical commercial toy with a small opening, filled with a mix of dry and wet food.	Food and Sensorial	Animal hits the toy with its nose or paws to turn the tool and make the food fall out.	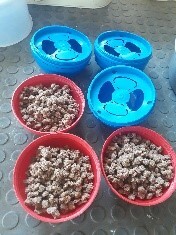
**Ice cream**	Wet food mixed with water in a blender and placed in an ice cube tray for freezing.	Food and Sensorial	Animal licks, bites, and/or ingests food.	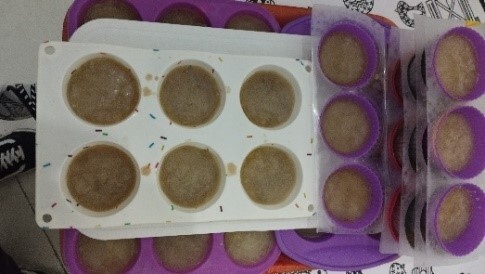
**Intraspecific**	Access to other animals of the same species, breed, and sex.	Social	Animal in direct physical contact with another animal, with parts of their body in contact with the other (mounting; nibbling; touching; “calling to play”; sniffing and nuzzling).	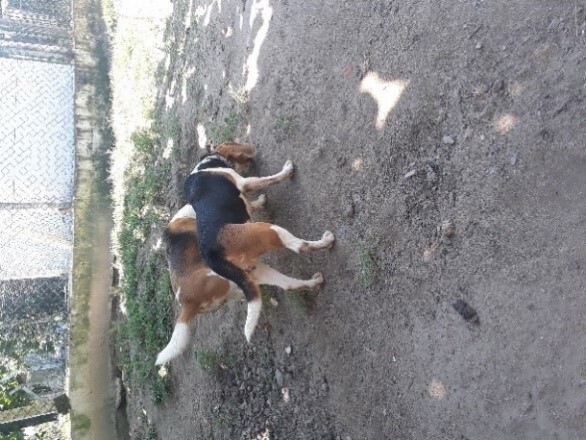
**Interspecific**	Access to human collaborator.	Social	Animal in direct physical contact with the team of collaborators, touching parts of their body (jumping on them; pawing, “calling to play”; sniffing and nuzzling).	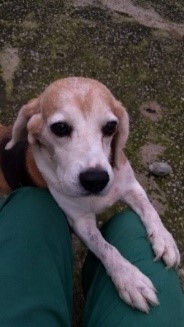
**Environmental interaction**	Access to a physical area other than the monotonous kennel.	Physical	Animal sniffing, urinating, defecating, running, jumping, vocalizing, and/or resting in the delimited area.	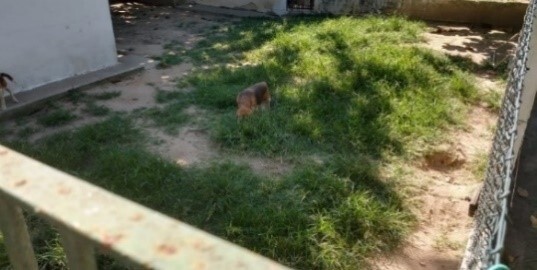

### Data collection

The evaluated data included the use of the pool with some PET bottle and interaction with the Pet Ball®, both involving the ingestion of a mix of dry and wet food concealed in the item; ingestion of wet food ice cream; interaction with another animal (sniffing, mounting, playing) and/or with the team member (sniffing, jumping on top, nuzzling, “asking for affection”); and use of the green area when demonstrating natural behaviors (running, sniffing, urinating, defecating, assembling, digging, and laying down).

The evaluations were conducted in the external area of the kennel. An ethogram (Table 2) was used to record the observations. Each tool had six squares that could be marked, with each square corresponding to every 5 min of observation, for a total period of 30 min (six moments) per day, one day per week, for a total of eight weeks. These observations were conducted in the afternoon, and a total of 48 interactions were evaluated. For example, square “5” corresponded to the first 5 min of observation. If animal 1 was interacting with the pool during this time, square “5” in the “pool” column was marked. If animal 2 was interacting with the ice cream during the 6-10 min of observation, box “10” in the “ice cream” column was marked.

**Table 2:** Ethogram identifying experimental animals, environmental enrichment tools used, and usage frequency during the experiment.

**DOGS**		**ENVIRONMENT ENRICHMENT TOOLS**																	
		**PET POOL**			**PETBALL**			**ICE CREAM**			**INTRA-ESPECÍFIC**			**INTER-ESPECÍFIC**			**ENV INTERACTION**		
**GROUP 1 FEMALES**	1	5	10	15	5	10	15	5	10	15	5	10	15	5	10	15	5	10	15
		20	25	30	20	25	30	20	25	30	20	25	30	20	25	30	20	25	30
	2	5	10	15	5	10	15	5	10	15	5	10	15	5	10	15	5	10	15
		20	25	30	20	25	30	20	25	30	20	25	30	20	25	30	20	25	30
	3	5	10	15	5	10	15	5	10	15	5	10	15	5	10	15	5	10	15
		20	25	30	20	25	30	20	25	30	20	25	30	20	25	30	20	25	30
**GROUP 2 MALES**	4	5	10	15	5	10	15	5	10	15	5	10	15	5	10	15	5	10	15
		20	25	30	20	25	30	20	25	30	20	25	30	20	25	30	20	25	30
	5	5	10	15	5	10	15	5	10	15	5	10	15	5	10	15	5	10	15
		20	25	30	20	25	30	20	25	30	20	25	30	20	25	30	20	25	30
	6	5	10	15	5	10	15	5	10	15	5	10	15	5	10	15	5	10	15
		20	25	30	20	25	30	20	25	30	20	25	30	20	25	30	20	25	30

**Note:** The ethogram consists of the identification of the animals participating in the experiment, the environmental enrichment tools used, and each time the animals chose to use them.

### Statistical analysis

Behavioral observations (activities) were evaluated over 8 days and initially analyzed to determine the nature of their distribution (parametric or not). The Shapiro-Wilk test was used for the analysis. The assessments were performed on the same animals at different time points. Based on the study design and the type of distribution found, statistical analyses were conducted for more than two paired samples. A parametric ANOVA for repeated measures was used to compare the average values of the behavioral observations in the different activities. The significance level was set at 95% (*p* ≤ 0.05). The statistical program used for the analysis was SPSS® (Field, 2020).

## Results

**Figure 1:**
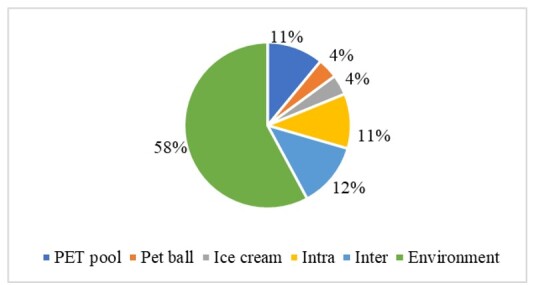
Use of EE tools by animals. Note: The graph displays the percentage of use of each environmental enrichment tool for all animals during the 8 d of the experiment.

The results, presented in Figure 1, indicate that the most used EE tool by the dogs, amounting to 58% use (*p* < 0.05), was environmental interaction in both groups of animals.

Social enrichment was assessed through the interaction of animals with each other and with the person present during the evaluation. As previously reported (Figure 1), there was a high occurrence of intra and interspecific behavior, totaling 23% and resulting in this second type of enrichment being the most used by the animals.

**Figure 2:**
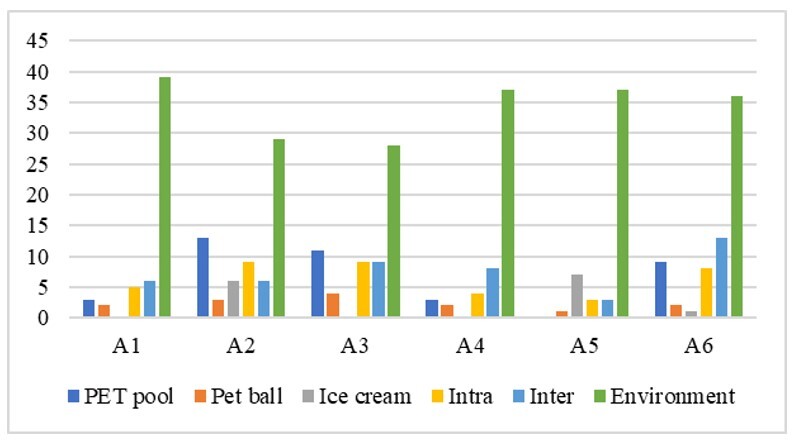
Interaction with EE tools for each animal. Note: The graph displays the absolute frequency of interaction of each animal with each EE tool offered during the 8 d of the experiment. A1 = Animal 1; A2 = Animal 2; A3 = Animal 3; A4 = Animal 4; A5 =Animal 5; A6 = Animal 6.

The use of tools specifically designed for food enrichment was the third most commonly used type of enrichment by the dogs (Figure 2). Occurrences were observed where the animals interacted with the Petball® toy, ice cream, and the PET bottle pool, with respective usage percentages of 4%, 4%, and 11%. It is important to note that all of these enrichment techniques were used with both dry and wet food.

**Figure 3:**
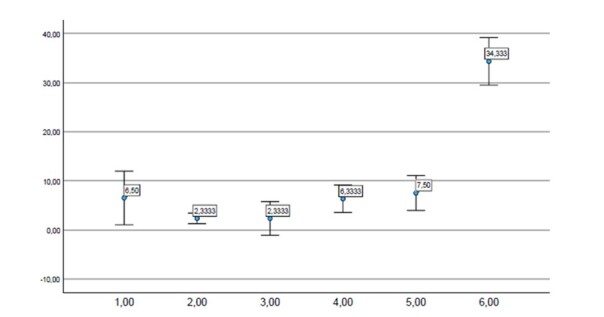
The graph shows the mean and standard deviation of the ratios between the 6 animals separated by each EE tool (1 = PET Pool; 2 = Petball; 3 = Ice Cream; 4 = Intra; 5 = Inter; 6 = Environment). *95% confidence interval.

No differences were observed between the other EE tools (Figure 3), such as Petball® and PET pool, ice cream and Petball®, or intra and inter socialization (*p* > 0.05). The only tool that showed high significance was the interaction with the environment.

## Discussion

The reason why the environmental interaction was chosen as the primary tool is probably because it provides an area of land and grass, which can be considered an environment with physical environmental enrichment. This enrichment is the result of structural modification of the animals’ living space (Hosey et al., 2009). It is also possible that this environment stimulated the animals’ preference for eliminating outside the kennel. Additionally, it was expected that the animals would engage in running and digging behaviors because of the deprivation of their normal behaviors and the size of the release area. Allowing the animals to demonstrate their natural behaviors, such as digging, eating grass, and running, is considered essential for maintaining their well-being (Young, 2003). This was further supported by a study conducted by the Duranton and Horowitz (2019), which found that dogs who participated in nosework treatment were “more optimistic” after the treatment compared to before.

In line with sensory enrichment, sniffing can also be considered an expected behavior. Figure 2 illustrates the frequency of each animal interacting with each type of EE tool. When entering new areas or areas that had not been used for some time, the dogs exhibited exploratory behavior. This is because these locations receive other dogs and retain traces of their distinct scents, favoring the high occurrence of sniffing. The new area consisted of dirt and grass, unlike the individual kennel made of cement. The dogs were also stimulated sensorially through touch. The sense of smell was not only stimulated by the new area, but also by the offering of ice cream, the PET bottle pool, and Petball® toys.

The success of a domestic dog as a species depends on their ability to interact socially with members of its own species, and the effectiveness of this interaction depends on both the repertoire of signals used to express social intentions and their cognitive ability to interpret the behavior of others (Serpel, 2017). When dogs are released into an area with other dogs, it has positive effects on their well-being, especially when they have been confined to individual kennels. The second type of enrichment preferred by dogs was social enrichment. Of all the common laboratory species, dogs are the most commonly socialized and adapted to living with humans. Socialization at a young age builds trust and attachment, which helps dogs cope with potentially stressful experiences in the future (Beaver, 1981).

Food enrichment, through the provision of toys, can help alleviate the monotony in confined environments (Campos et al., 2010). This is important because experiencing boredom can be aversive and may lead to issues such as depression-like states or self-injurious behavior if not addressed (Meagher, 2019). During observations, it was noted that animals interacted with the Petball® toy, ice cream, and the PET bottle pool. These techniques involved a mix of dry and wet food to stimulate the animals’ interest. The use of tools specifically designed for food enrichment was the third most commonly used type of enrichment by dogs.

Hubrecht (1995) recommends suspending toys from the floor to prevent fights caused by possessiveness and to keep them clean. This recommendation was supported by the findings of the present study, wherein some dogs exhibited possessive behavior towards the Petball® toy even though it was placed in one unit for each animal. However, in this study, the toys were not suspended.

The feeding behavior of dogs is not only related to the act of consuming food, but also to the time spent in the process. Dogs spend a certain amount of time per day eating, and this can be significantly extended through environmental food enrichment strategies that keep the animal active for a longer period of time, preventing monotony and reducing stress. Providing concealed food encourages dogs to explore their surroundings while engaging their nose, mouth, and paws simultaneously (Horowitz, 2010). This was demonstrated in this study using a pool of PET bottles containing hidden dry and wet food.

## Conclusions

These findings indicate that dogs housed in individual kennels were able to utilize the physical environmental enrichment tool to engage in their natural behaviors. This suggests that their overall well-being and likely their mental health were maintained at a satisfactory level.
